# Pathogenic and genomic characterisation of a rabbit sourced *Pasteurella multocida* serogroup F isolate s4

**DOI:** 10.1186/s12917-022-03381-7

**Published:** 2022-07-23

**Authors:** Jinxiang Wang, Shikun Sun, Yanfeng Chen, Dongjin Chen, Lei Sang, Xiping Xie

**Affiliations:** Institute of Animal Husbandry and Veterinary Medicine, Fujian Academy of Agricultural Sciences, No. 100 Pudang Road, Xindian Town, Jin’an District, Fuzhou, Fujian 350013 People’s Republic of China

**Keywords:** *Pasteurella multocida* serogroup F, Rabbit, Pathogenicity, Genomic feature

## Abstract

**Background:**

*Pasteurella multocida* is one of the most significant pathogens for a number of animals. In rabbits, the infection is generally associated with the *P. multocida* serogroups A and D, and the knowledge about the serogroup F is limited. In the present study, a *P. multocida* serogroup F isolate designated s4 was recovered from the lungs of rabbits died of respiratory disease in Fujian, in the southeast of China. The pathogenicity and genomic features of the s4 were then determined.

**Results:**

The serotype and sequence type of s4 were F:L3 and ST12, respectively. The s4 was pathogenic for rabbits, but it was a low virulent strain comparing to the previously reported highly pathogenic *P. multocida* serogroup F strains J-4103, C21724H3km7, P-4218 and HN07. The whole genome of the s4 was then sequenced to understand the genomic basis for pathogenicity. Particularly, a large-sized fragment of approximate 275 kb in length was truncated from the chromosome to form a plasmid. Moreover, the in-frame deletion of *natC* and N-terminal redundance of *gatF* would resulted in the production of a mutant L3 outer core structure that was distinct from those of the other *P. multocida* strains belonging to the lipopolysaccharide genotype L3. We deduced that these features detected in the genome of s4 might impair the pathogenicity of the bacterium.

**Conclusions:**

This study evaluated the pathogenicity and determined the genomic features of the rabbit sourced *P. multocida* serogroup F isolate s4, the observations and findings would helpful for the understanding of the pathogenicity variability and genetic diversity of *P. multocida*.

**Supplementary Information:**

The online version contains supplementary material available at 10.1186/s12917-022-03381-7.

## Background

*Pasteurella multocida* is recognized as one of the important pathogens for a wide range of animals. The infection of *P. multocida* is often associated with economically important diseases, such as fowl cholera in poultry, haemorrhagic septicaemia in bovine, progressive atrophic rhinitis in swine, and respiratory disease in rabbit [1, 2]. *P. multocida* strains can be classified into five capsular serogroups or capsular genotypes (A, B, D, E and F) and further classified into sixteen lipopolysaccharide (LPS) serotypes (1–16) or eight LPS genotypes (L1-L8) [3, 4]. *P. multocida* strains can also be typed by using the multi-locus sequence typing (MLST) scheme based on the seven housekeeping genes [5, 6]. Rabbit pasteurellosis is generally associated with the strains of capsular serogroups A and D, LPS genotypes L3 and L6, and MLST sequence types ST10, ST11 and ST12 [7, 8]. However, some reports showed that strain of serogroup F that predominately circulated in poultry had also been detected in rabbits, and the bacterium was highly pathogenic for rabbits [9–12].

Rabbit farming is popular in Fujian, in the southeast of China [8, 13]. The number of rabbits farmed and the amount of rabbit meat yielded in Fujian were about 10.67 million and 16.2 thousand tons by the end of 2019 (Fujian Statistical Yearbook, 2020), respectively. Our previous work showed that *P. multocida* was widespread in rabbits in Fujian, but only strains of serogroups A and D were detected [8]. In July 2020, a respiratory infectious disease broke out on a local rabbit farm in Fujian, and around 500 rabbits died of the disease during a 30-day period. Clinical signs of cough and nasal discharge were observed in the diseased rabbits. Gross pathological lesions including hemorrhagic pneumonia and pulmonary consolidation were showed in the dead rabbits. Four PCR assays were used to screen the potential causative agents including Rabbit hemorrhagic disease virus (RHDV) [14], *P. multocida* [3], *Bordetella bronchiseptica* (*B. bronchiseptica*) [15] and *Staphylococcus aureus* (*S. aureus*) [16] in the 48 lung samples collected from the dead rabbits. The results showed that the RHDV, *B. bronchiseptica* and *S. aureus* were negative, but the *P. multocida* was positive for the all 48 lung samples and a *P. multocida* serogroup F isolate named s4 was recovered from the all samples. To our knowledge, the information on the isolation and/or identification of *P. multocida* serogroup F strain in the rabbits in Fujian is limited, and especially, there was no report concerning the pathogenicity of rabbit sourced *P. multocida* serogroup F strain isolated from Fujian.

In present study, the s4 was defined by capsular typing, LPS genotyping, MLST, screened virulence factors, and evaluated the pathogenicity in rabbits. Moreover, the whole genome sequence of s4 was determined, and the whole genome comparison between the s4 and the other *P. multocida* serogroup F strains was performed. The aim of the present study was to evaluate the pathogenicity and determine the genetic features of the rabbit sourced *P. multocida* serogroup F isolate s4.

## Results

### *P. multocida* isolation and identification

The presence of the potential pathogens including RHDV, *P. multocida*, *B. bronchiseptica* and *S. aureus* in the 48 lung samples collected from the natural infected dead rabbits were determined by using PCR assays. The results showed that RHDV, *B. bronchiseptica* and *S. aureus* were negative for the all 48 lung samples, whereas *P. multocida* was positive for the all 48 lung samples. All the lung samples were then subjected to *P. multocida* isolation.

All the 48 lung samples produced identical bacterial colonies on brain heart infusion (BHI) blood agar plate. The colonies were grey, round, smooth and less than 1 mm in diameter. Three colonies from each lung sample were randomly picked up. The sequences of the 16S rRNA genes of the 144 isolates recovered from the 48 lung samples were identical and shared the highest identity (range from 99.90 to 100%) with that of *P. multocida*. The PCR assays for capsular and LPS typing of the isolates yielded segments of approximate 850 bp and 470 bp in length, and the sequences of the two segments were 100% identical with the *fcbD* and *gatF* of *P. multocida*, respectively. The results indicated that the 144 isolates were *P. multocida* serotype F:L3.

### MLST

The allelic numbers of the 7 housekeeping genes (*adk*, *aroA*, *deoD*, *gdhA*, *g6pd*, *mdh* and *pgi*) of the 144 isolates were identical and assigned as 8–8–5-3-5-7-5, and the sequence types of the 144 isolates were then defined as ST12.

### Virulence genes detection

The virulence genes *ptfA*, *tadD*, *hgbB*, *ompA*, *omph* and *oma87* were positive for the 144 isolates, whereas the virulence genes *pfhA*, *toxA*, *fur*, *tbpA*, *nanB* and *pmHAS* were negative for the all isolates. The sequence of each of the virulence genes (*ptfA*, *tadD*, *hgbB*, *ompA*, *omph* and *oma87*) of the 144 isolates were identical, and the sequences of these six virulence genes shared highest identity (ranged from 99.90 to 100%) with the corresponding virulence genes of *P. multocida*.

**Fig. 1:**
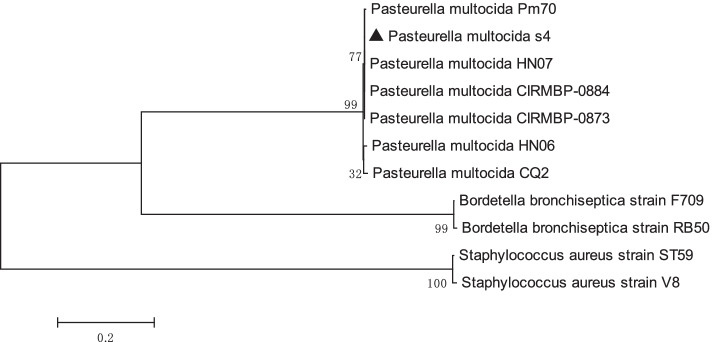
Neighbour-joining tree indicating the position of *P. multocida* isolate s4 based on the seven MLST genes. The seven MLST genes of *P. multocida* (*adk*, *aroA*, *deoD*, *gdhA*, *g6pd*, *mdh* and *pgi*), *B. bronchiseptica* (*adk, fumC, glyA, tyrB, icd, pepA and pgm*) and *S. aureus* (*arcC, aroE, glpF, gmk, pta, tpi and yqiL*) were concatenated, and then the neighbour-joining tree was constructed using MEGA 5.0 (1000 of bootstrap replications). The senve MLST genes of the strains were obtained under the accession numbers: *P. multocida* strains Pm70 (AE004439), HN07 (CP007040), CIRMBP-0884 (CP020345), CIRMBP-0873 (CP020347), HN06 (CP003313) and CQ2 (CP033599); *B. bronchiseptica* strains F709 (CP020818) and RB50 (BX470250); *S. aereus* strains ST59 (CP076823) and V8 (CP079715)

Taken together, the serotypes, sequence types and the virulence gene profiles of the 144 isolates were identical, suggesting that all the isolates were derived from the same progenitor (Fig. 1). In this study, one of the isolates named s4 was selected as the representative for the following animal experiments and whole genome sequencing.

### Animal experiments

**Fig. 2:**
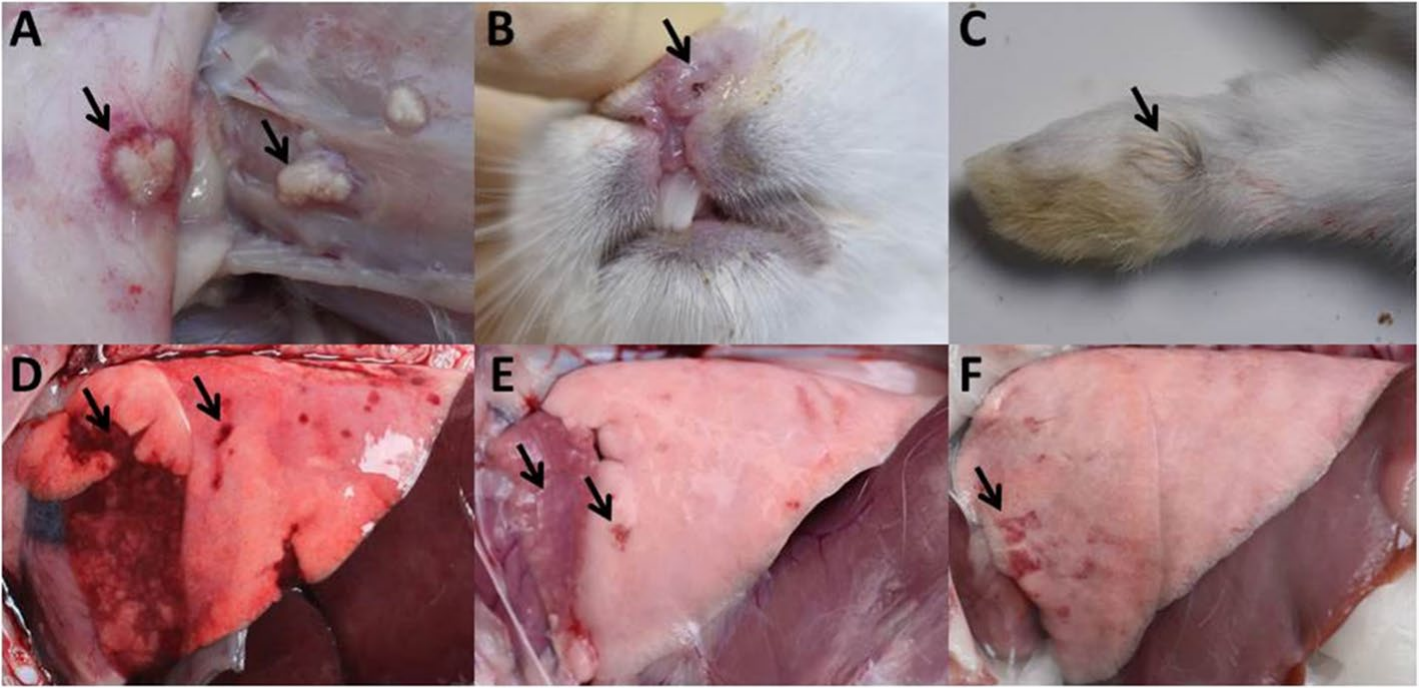
Clinical signs and gross lesions of the rabbits challenged with s4. **A** Local subcutaneous abscess of the rabbits subcutaneously infected with 6.0 × 10^4^ CFU of the s4; **B** Nasal discharge of the rabbit intranasally infected with 6.0 × 10^4^ CFU of the s4; **C** Matted forepaws of the rabbits intranasally infected with 6.0 × 10^4^ CFU of the s4; **D** Hemorrhagic pneumonia of the rabbit intranasally infected with 6.0 × 10^4^ CFU of the s4; **E** Pulmonary consolidation and weak hemorrhagic pneumonia of the rabbits intranasally infected with 6.0 × 10^4^ CFU of the s4; **F** Weak hemorrhagic pneumonia of the rabbits intranasally infected with 6.0 × 10^4^ CFU of the s4

**Fig. 3:**
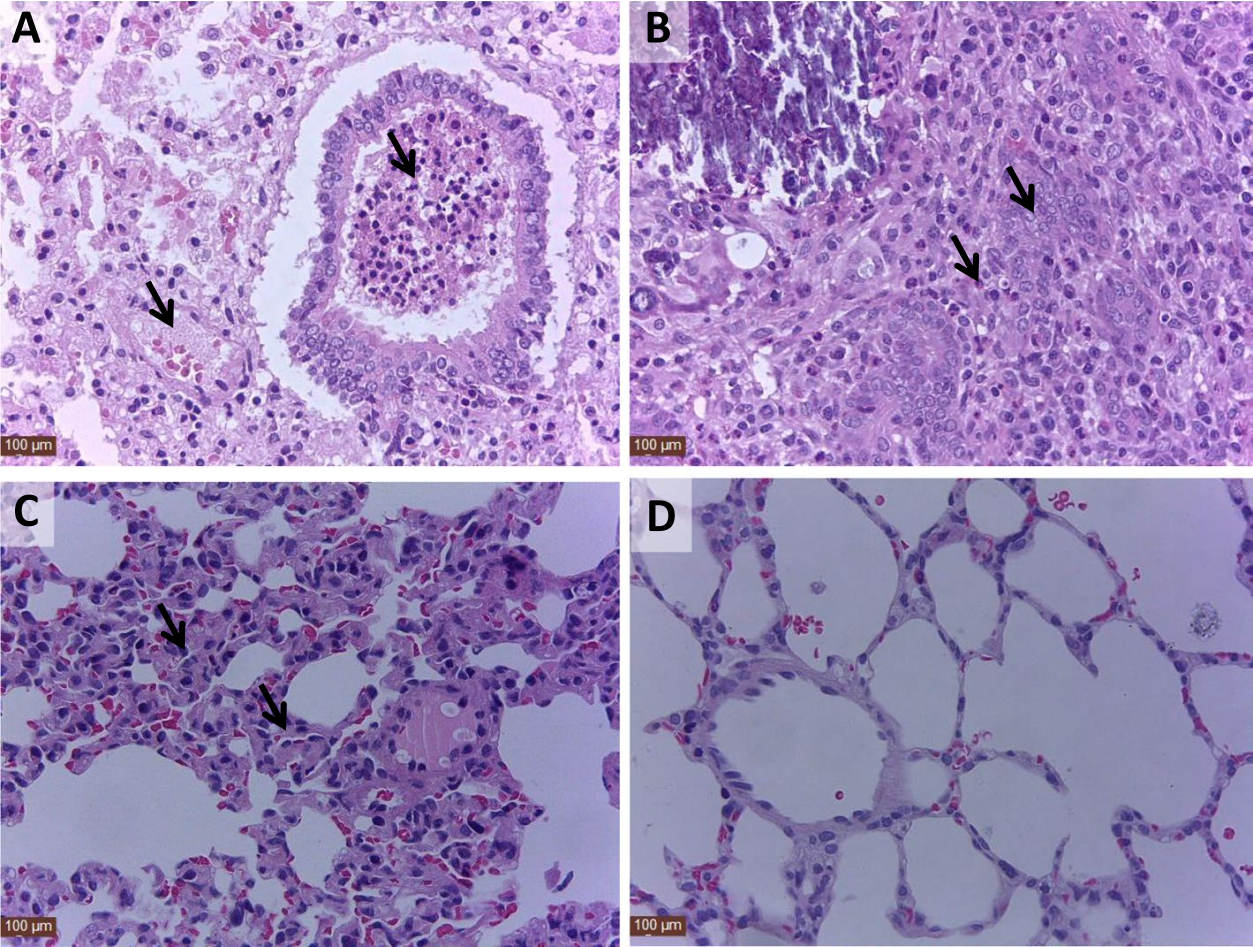
Histological lesions in the lungs of the rabbits intranasally infected with 6.0 × 10^4^ CFU of the s4 (hematoxylin-eosin staining, × 400). **A** Inflammatory exudates appeared in the bronchiole and alveoli as well as degeneration of the alveolar epithelial cells in the one rabbit with hemorrhagic pneumonia; **B** Inflammatory exudates in the bronchiole and alveoli as well as proliferation of alveolar epithelial cells in the two rabbits with pulmonary consolidation and weak hemorrhagic pneumonia; **C** Proliferation of alveolar epithelial cells in the five rabbits with weak hemorrhagic pneumonia; **D** The lung tissues from the rabbits of negative control group

To evaluate the virulence of *P. multocida* serogroup F isolate s4 in rabbits, groups of animals were subcutaneously or intranasally challenged with 6.0 × 10^4^ colony forming units (CFU) of the s4. All the eight rabbits in the subcutaneous inoculation group survived the challenge, and all of the rabbits showed no clinical signs of disease. The only gross lesion of local subcutaneous abscess at the inoculation site was observed in the all rabbits from the subcutaneous inoculation group (Fig. 2A), and the tissue samples including tracheas, lungs, livers, hearts, spleens, kidneys and blood of the all rabbits from the subcutaneous inoculation group were negative for *P. multocida*. One rabbit in the intranasal inoculation group developed distinct clinical signs of cough and nasal discharge on 6 days post challenge (Fig. 2B), and the rabbit was caught up in the endpoint on 15 days post challenge because of inability to access feed and water. However, the only clinical sign of matted forepaws was showed in the remaining 7 rabbits in the intranasal inoculation group (Fig. 2C), and the seven rabbits survived the challenge. Gross lesions including hemorrhagic pneumonia (one rabbit) (Fig. 2D), pulmonary consolidation and weak hemorrhagic pneumonia (two rabbits) (Fig. 2E), and weak hemorrhagic pneumonia (five rabbits) (Fig. 2F) were observed in the rabbits in the intranasal inoculation group. The s4 was re-isolated from the following tissue samples collected from rabbits in the intranasal inoculation group: tracheas (*n* = 8/8), lungs (*n* = 8/8), livers (*n* = 8/8), hearts (*n* = 0/8), spleens (*n* = 0/8), kidneys (*n* = 0/8) and blood (*n* = 0/8). Histologically, inflammatory exudates appeared in the bronchiole and alveoli as well as degeneration of the alveolar epithelial cells were observed in the lung of the rabbit with hemorrhagic pneumonia (Fig. 3A), inflammatory exudates in the bronchiole and alveoli as well as proliferation of alveolar epithelial cells were shown in lungs of the two rabbits with pulmonary consolidation and weak hemorrhagic pneumonia (Fig. 3B), only proliferation of alveolar epithelial cells was shown in lungs of the five rabbits with weak hemorrhagic pneumonia (Fig. 3C). Interestingly, all the eight rabbits in the subcutaneous inoculation group and five rabbits in the intranasal inoculation group were serological positivity for *P. multocida* IgG on 15 days post challenge. During the 15-day experimental period, all the rabbits in the control group remained *P. multocida* free, and showed no clinical signs and gross lesions of disease.

### Genome sequencing and comparative analysis

**Fig. 4:**
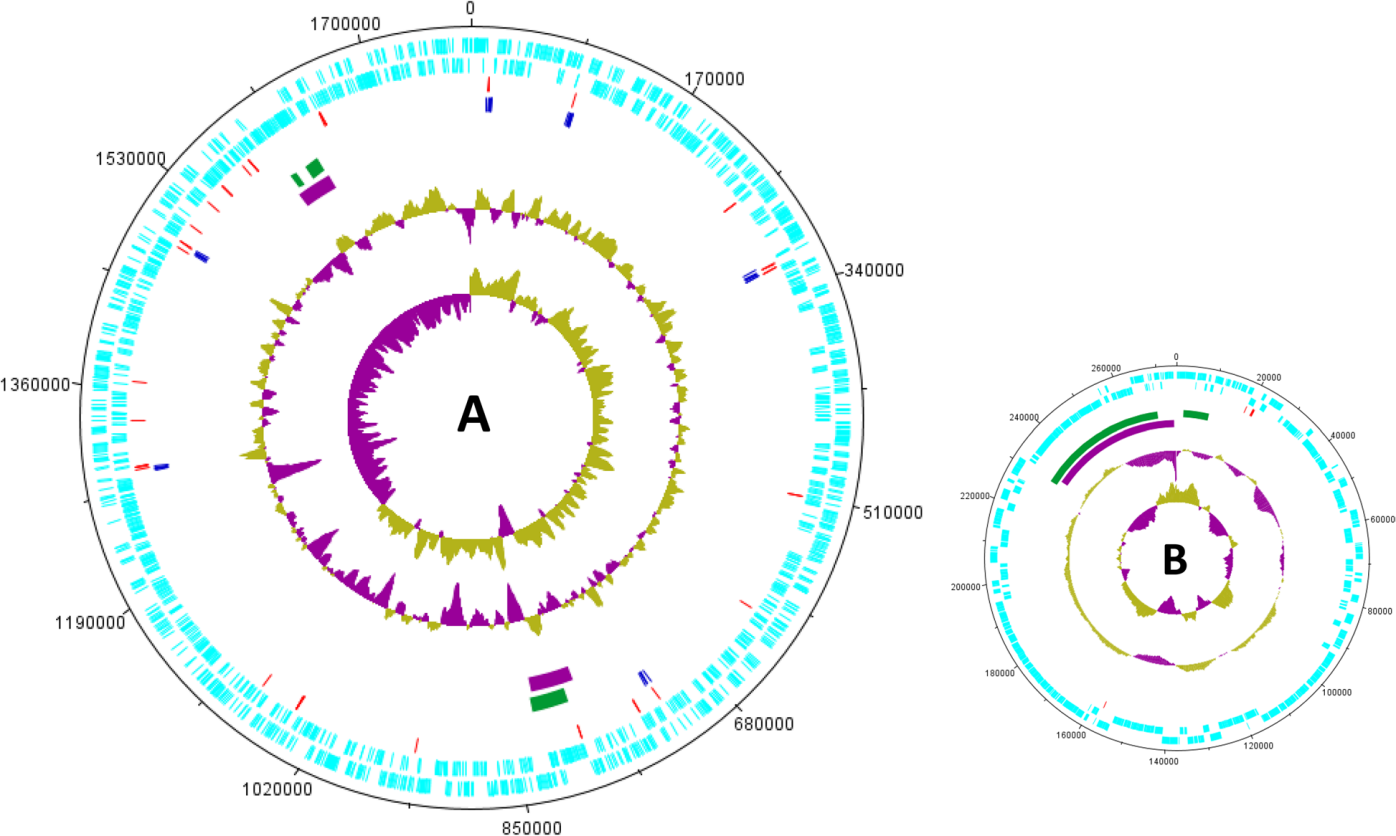
Circular maps of the s4 genome generated by using DNAPlotter. **A** The chromosome of s4; **B** The plasmid of s4. From the outside to the inside, circle 1 (black): DNA base position; circle 2 (cyan-blue): protein-coding regions in forward strand; circle 3 (cyan-blue): protein-coding regions in reverse strand; circle 4 (red): tRNA genes; circle 5 (blue): rRNA genes; circle 6 (green): genomic islands; circle 7 (purple): prophages; the two innermost circles represent the G + C content and GC skew, respectively

**Fig. 5:**
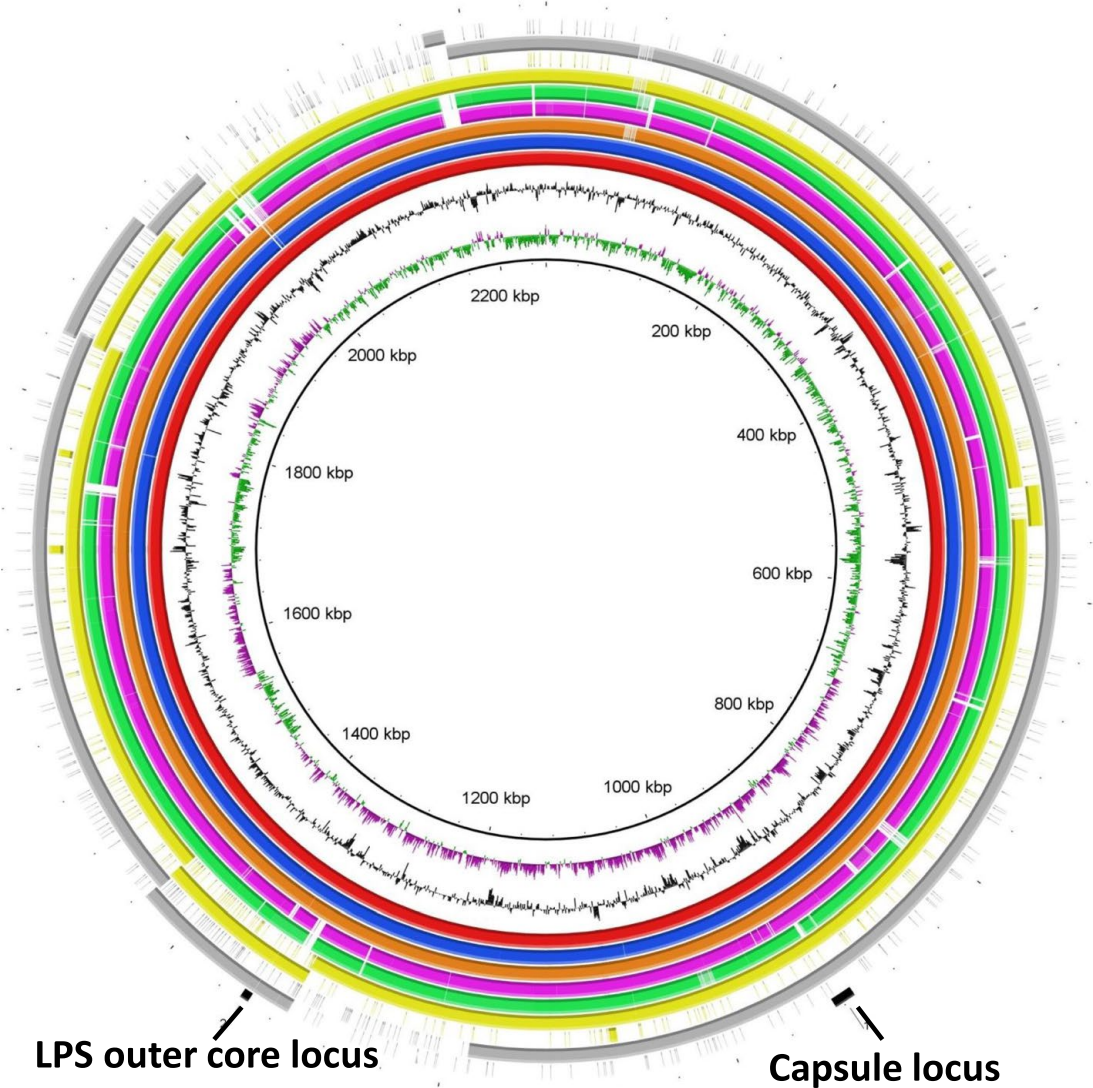
Comparation between the genome of s4 and those of CIRMBP-0884, HN06, CQ2, CIRMBP-0873, HN07 and Pm70 at nucleotide level. From the outside to the inside, circle 1 (black): positions of capsule locus and LPS outer core locus; circle 2 (gray): plasmid of s4; circle 3 (gray): chromosome of s4; circle 4 (yellow): plasmid of CIRMBP-0884; circle 5 (yellow): chromosome of CIRMBP-0884; circle 6 (green): CQ2; circle 7 (purple): HN06; circle 8 (orange): CIRMBP-0873; circle 9 (blue): HN07; circle 10 (red): Pm70; circles 11 and 12 represent the G + C content and GC skew, respectively; the innermost circle represents DNA base position

The complete genome of the s4 was approximately 2.06 Mbp in length, which is 201,495 bp smaller than that of avian sourced *P. multocida* serogroup F strain Pm70 [17]. Interestingly, the complete genome of s4 consisted of a chromosome and a plasmid. The complete genome of the s4 has been deposited to the NCBI GenBank database under the accession numbers of CP084165 (chromosome) and CP084164 (plasmid). The chromosome of s4 was 1,781,242 bp in length with the G + C content of 40.42%, in which 1619 protein coding sequences, 45 tRNA genes, 19 rRNA genes, 3 Genomic islands (GIs) and 2 intact prophages were determined (Fig. 4A). The plasmid of s4 was 274,750 bp in length with the G + C content of 39.62%, in which 302 protein coding sequences, 7 tRNA genes, 2 GIs and one intact prophage were determined (Fig. 4B). Interestingly, BLAST analysis showed that the plasmid of s4 was highly homologous (up to 85% identity) with the other *P. multocida* genomes at nucleotide level (Fig. 5). By blasting against the Comprehensive Antibiotic Resistance Database (CARD), two elfamycin resistance genes and one cephalosporin resistance gene were predicted, and all of the three drug resistance genes were located in the chromosome of s4.

**Fig. 6:**
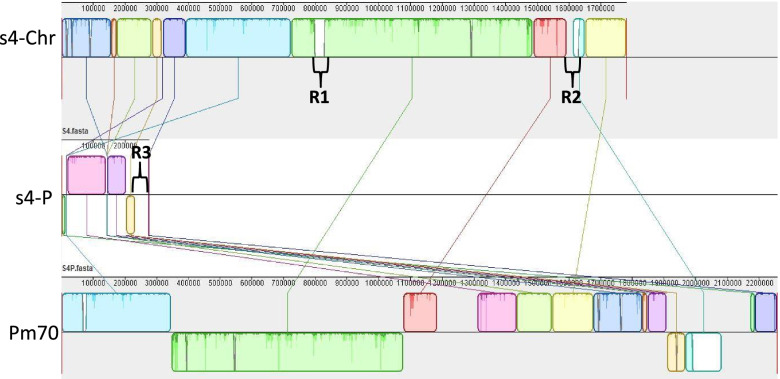
Colinear analyses between the genomes of s4 and Pm70. s4-Chr and s4-P represent the chromosome and plasmid of s4, respectively. Rectangle of the same colour indicates the similar local colinear block of the s4 and Pm70. R1, R2 and R3 indicate the three large regions of specific sequences in the genome of s4 but not in that of Pm70

Phylogenetic analysis showed that the s4 was closely related to the well- characterized avian sourced *P. multocida* serogroup F strain Pm70 (Fig. 1) [17]. Therefore, the genome of s4 was compared with that of Pm70. By comparing with the genome of Pm70, gene rearrangement, gain and loss were observed in that of s4. Especially, three large regions (R) of specific sequences were found in the genome of s4 (Fig. 6). Two regions (R1 and R2) located in the chromosome and were approximately 31.9 kb and 37.0 kb in length, respectively. The third region (R3) located in the plasmid and was approximately 42.3 kb in length. Interestingly, all of the three regions located in the intact phage regions that were determined in s4 genome by using PHAST [18]. Additionally, one unique gene (LCY73_01710) encoding the TonB-dependent receptor was identified in the genome of s4 but absent in that of Pm70.

**Fig. 7:**
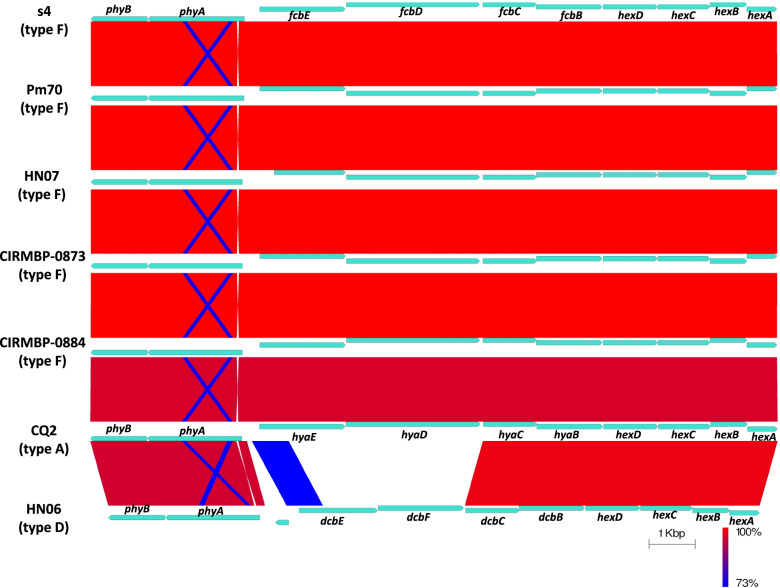
Comparative analyses of the entire *cap* locus between the s4 and other *P. multocida* strains. The entire *cap* locus of the s4 were compared with those of Pm70 (capsular type F), HN07 (capsular type F), CIRMBP-0873 (capsular type F), CIRMBP-0884 (capsular type F), CQ2 (capsular type A) and HN06 (capsular type D). The color code represents the BLASTn identity

The entire *cap* locus of s4 were compared with those of Pm70 (capsular type F), HN07 (capsular type F), CIRMBP-0873 (capsular type F), CIRMBP-0884 (capsular type F), CQ2 (capsular type A) and HN06 (capsular type D). The result showed that the entire *cap* locus of s4 was highly matched with those of capsular type F strains (Pm70, HN07, CIRMBP-0873 and CIRMBP-0884), and the entire *cap* locus of s4 was closely matched with that of capsular type A strain (CQ2) but was distinct from that of capsular type D strain (HN06) (Fig. 7).

**Fig. 8:**
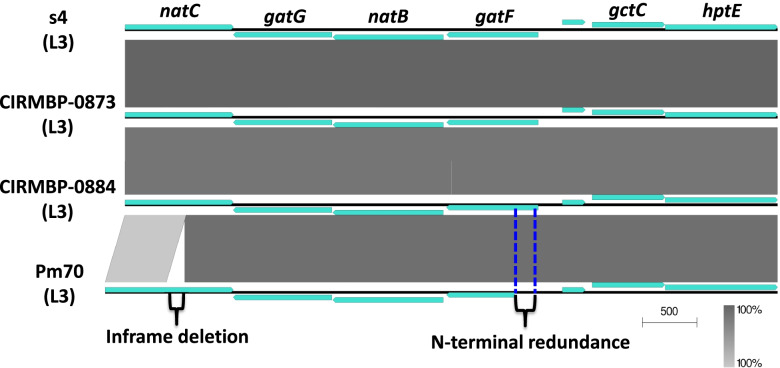
Comparative analyses of the LPS outer core locus of s4. The genes required for the assembly of the LPS outer core of s4 were compared with those of CIRMBP-0873 (LPS genotype L3), CIRMBP-0884 (LPS genotype L3) and Pm70 (LPS genotype L3). The color code represents the BLASTn identity

The LPS outer core biosynthetic genes of s4 were compared with that of Pm70. All of the LPS outer core biosynthetic genes of Pm70 were located in the chromosome, whereas these genes were located in the plasmid of s4. With the exception of *natC* and *gatF*, the LPS outer core biosynthetic genes of s4 including *gatG*, *natB*, *gctC* and *hptE* were highly matched with those of Pm70. Interestingly, an in-frame deletion of 183-bp nucleotide (in positions 559–741) was observed in the *natC* of s4, which resulted in a deletion of 61 amino acids (in positions 183–243) of the glycosyltransferase NatC of s4 by comparison with that of Pm70 (Fig. 8). Additionally, the *gatF* of s4 possessed a 210-bp nucleotide sequence redundance at the N-terminal by comparison with that of Pm70 (Fig. 8).

## Discussion

*P. multocida* serogroup F was first isolated from turkeys in the USA in 1987 [19], and since then it has been mainly associated with the infections in avian hosts [20–22]. *P. multocida* is one of the important pathogens results in morbidity and mortality in rabbits, whereas only the serogroups A and D had been previously recognized as the causative agents of rabbit pasteurellosis [7, 12]. It was not until 2008 that the pathogenicity of *P. multocida* serogroup F for rabbit was documented [12]. Since then, the presence of *P. multocida* serogroup F has been identified in the important rabbit farming areas worldwide [9–11]. However, the knowledge about the pathogenicity and genetic characteristics of the rabbit sourced *P. multocida* serogroup F is still limited. In the present study, the pathogenicity of a rabbit sourced *P. multocida* serogroup F isolate s4 was evaluated and the whole genome of the isolate was sequenced, which would helpful for the understanding of the pathogenitity of rabbit sourced *P. multocida* serogroup F.

Unexpectedly, the inoculation of s4 did not cause death and result in severe gross lesions in most of the challenged rabbits. The results were in contradict with the previous study, in which the inoculation of rabbit sourced *P. multocida* serogroup F isolate J-4103 caused high mortality and resulted in severe pathological lesions in most of the challenged rabbits (acute septicemic syndrome and extensive hemorrhage in subcutis in subcutaneously challenged rabbits, and fibrinopurulent pleuropneumonia and extensive hemorrhagic pneumonia in intranasally challenged rabbits) [12]. Moreover, *P. multocida* serogroup F isolates from chicken (C21724H3km7), turkey (P-4218) and pig (HN07) were also highly virulent to rabbit without prior adaption [23, 24]. The results suggested that the variation in virulence among *P. multocida* serogroup F strains, and the s4 was a low virulent strain by comparison with the J-4103, C21724H3km7, P-4218 and HN07. Additionally, it should be alert to the potential transmission of *P. multocida* serogroup F from chicken, turkey and pig into rabbit, which might cause high mortality and subsequent huge economic loss to rabbit farming.

*P. multocida* possesses a number of virulence genes, which contributes to the fitness and pathogenicity of the pathogen [1]. The s4 carried a panel of virulence genes including *ptfA*, *tadD*, *hgbB*, *ompA*, *omph* and *oma87*. The *ptfA*, *tadD*, *ompA*, *omph* and *oma87* encode the proteins that are associated with *P. multocida* virulence because of their roles in the attachment and colonization of host [25, 26]. The *hgbB* encodes an outer membrane protein, which is involved in iron acquisition [25]. Interestingly, the *P. multocida* serogroup D isolates carrying the *hgbB* were significantly associated with the clinical presentation of respiratory disease in rabbits [9]. Additionally, comparative genomic analyses between s4 and Pm70 identified a unique gene in s4 but absent in Pm70. The unique gene (LCY73_01710) encoded TonB-dependent receptor that is essential for transport of organic iron chelators (siderophores) into the periplasm to establish commensal and pathogenic relationships with the hosts [27]. In Pm70, more than 2.5% of the genome was devoted to genes encoding proteins involved in iron uptake and acquisition, suggesting that iron uptake and acquisition is important for *P. multocida* survival and pathogenesis [17]. Taken together, the presence of these genes might contribute to the pathogenicity of the s4.

The complete genome of s4 was sequenced and then the comparative genomic analyses were performed to better understand the genetic basis for the pathogenicity of s4. The genome of s4 shared a high level of DNA identity to that of Pm70. However, the genome of s4 was smaller (approximate 200 kb) than that of Pm70, which might result in the loss of a number of functional genes and subsequently impaired the pathogenicity of the s4. Interestingly, the s4 possessed a plasmid of approximate 275 kb in length, and the sequence of the plasmid shared a high level of DNA identity (up to 90% identity) to the plasmid (approximate 325 kb in length) of rabbit sourced *P. multocida* serogroup F strain CIRMBP-0884. Additionally, the plasmids of the rabbit sourced *P. multocida* serogroup F strains s4 and CIRMBP-0884 were highly identical (up to 85% indentity) to the other *P. multocida* genomes at nucleotide level. Therefore, it was deduced that the sequences of the plasmids of s4 and CIRMBP-0884 might derive from their own genomes. Interestingly, only a small number of *P. multocida* strains possess plasmids according to the *P. multocida* genomes deposited in the NCBI genome database. The plasmids of *P. multocida* strains often carry antibiotic resistance genes and mobilization genes [28], and plasmids of some *P. multocida* strains carry the *toxA* gene encoding the dermonecrotic toxin [28]. Unexpectedly, the antibiotic resistance genes, mobilization genes and toxin gene were not determined in the plasmid of s4.

LPS is an important virulence determinant of *P. multocida* [29]. The LPS of the s4 belongs to the genotype of L3. It was showed that *P. multocida* strains belonging to the LPS genotype L3 might be highly virulent because these strains were the most common causative agents of fowl cholera [4, 29]. The L3 outer core structure is similar to the self-antigen of host, which facilitates the bacteria evading the host innate immune system [29]. The in-frame deletion of *natC* and the N-terminal redundance of *gatF* would result in the production of a mutant L3 outer core structure, which might contribute to the low pathogenicity of s4. Interestingly, the genetic diversities of *natC* and *gatF* from *P. multocida* serogroup F:L3 strains of CIRMBP-0873, CIRMBP-0884 and HN07 were also detected (Fig. 8) [30]. Taken together, the effects of the polymorphism in the LPS outer core genes *natC* and *gatF* on the pathogenicity of *P. multocida* would be worth eliciting in the further work.

## Conclusion

In the present study, the pathogenic and genetic characteristics of a rabbit sourced *P. multocida* serogroup F isolate s4 were determined. This study revealed that the s4 was low pathogenicity for rabbits by comparison with the previously reported highly virulent *P. multocida* serogroup F isolates J-4103, C21724H3km7, P-4218 and HN07, which was helpful for the awareness of the pathogenicity variation among *P. multocida* serogroup F isolates. Besides, the complete genome of the s4 was sequenced. The obtaining of its genomic sequence and features would provide a new genome for the *P. multocida* genome database. Particularly, the identification of natural truncation of large-sized sequence from genome to form a plasmid, and the in-frame deletion and N-terminal redundance of the LPS outer core assembly genes would helpful for understanding the genetic diversity of *P. multocida*.

## Methods

### Sample collection and detection of potential pathogen

A total of 48 lung samples were collected from the naturally infected dead rabbits. Each lung sample was placed in a sterile tube, kept on ice and delivered to our lab for examination within 24 h. Each lung sample was homogenized to make a 50% suspension in sterile normal saline. For the detection of RHDV, the suspension was centrifuged at 1500×g for 15 min at 4 °C, and two hundred microliter of the supernatant was collected and used for viral RNA extraction using the *EasyPure* Viral DNA/RNA kit (TransGen Biotech, Beijing, China). The extracted viral RNA was reverse-transcribed to complementary DNA (cDNA) using the *EasyScript* Reverse Transciptase (TransGen Biotech, Beijing, China) by using the Oligo-dT_18_ as the primer. The synthetic cDNA was used as the genomic template to screen the presence of the RHDV in the lung samples by using the PCR assay described by Schwensow et al. [14]. For the detection of *P. multocida*, *B. bronchiseptica* and *S. aureus*, one hundred microliter of lung suspension was centrifuged at 5000×g for 10 min at room temperature, and the pellet was used for bacterial DNA extraction using the *EasyPure* Bacteria Genomic DNA kit (TransGen Biotech, Beijing, China). The extracted DNA was used as the genomic template to screen the presence of the *P. multocida*, *B. bronchiseptica* and *S. aureus* in the lung samples by using the PCR assays described by Townsend et al. [3], Wang et al. [15] and Brakstad et al. [16], respectively.

### *P. multocida* isolation and identification

One hundred microliter of the lung suspension was spread on the BHI agar plate containing 5% defibrinated sheep blood and incubated at 37 °C for 24 to 48 h. Three isolates from each plate were randomly picked up and each isolate was inoculated in 5 mL of BHI containing 2% bovine serum. Then, the isolates were shaken at the conditions of 180 rpm and 37 °C for 24 h. To confirm the identities of the isolates, the 16S rRNA genes of the isolates were amplified and sequenced [31], and the capsular types and LPS genotypes of the isolates were defined using the PCR assays described by Townsend et al. [3] and Harper et al. [4], respectively.

### MLST

The MLST analyses of the isolates were conducted using the Multi-host MLST scheme described in the PubMLST (https://pubmlst.org/pmultocida/). The genomic DNA of the isolates were prepared using the *EasyPure* Bacteria Genomic DNA kit (TransGen Biotech, Beijing, China), and the seven housekeeping genes including *adk*, *aroA*, *deoD*, *gdhA*, *g6pd*, *mdh* and *pgi* of each isolate were amplified from the genomic DNA of the isolates. The expected PCR products were purified and subjected to sequence. The allelic profile of each isolate was defined by comparing the sequences of the 7 housekeeping genes of each isolate to the corresponding known sequences in the PubMLST database. The sequence type of each isolate was then defined by submitting the allelic profile to the PubMLST database.

### Virulence genes detection

The genomic DNA of the isolates were prepared using the *EasyPure* Bacteria Genomic DNA kit (TransGen Biotech, Beijing, China) and were screened for the presence of twelve virulence genes including adhesion related proteins (*ptfA*, *tadD* and *pfhA*), dermonecrotoxin (*toxA*), iron binding proteins (*fur*, *tbpA* and *hgbB*), sialidases (*nanB*), hyaluronidase (*pmHAS*) and outer membrane proteins (*ompA*, *ompH* and *oma87*). Primers used for *ptfA*, *tadD*, *pfhA*, *toxA*, *fur*, *nanB*, *pmHAS*, *ompA*, *ompH* and *oma87* were described by Tang et al. [26]. Primers used for *tbpA* and *hgbB* were described by Ewers et al. [25]. The expected PCR products were purified and sequenced, and the identities of these products were confirmed by comparing the sequences against the NCBI GenBank database.

### Animal experiments

Twenty-four 30-day-old rabbits obtained from a local rabbit farm were randomly divided into three groups (subcutaneous inoculation group, intranasal inoculation group and negative control group) of eight rabbits each (four males and four females). Each group was placed in a separate room, and two rabbits (one male and one female) from the same group were kept in a cage. Before infection, nasal, conjunctival and rectal swabs as well as whole blood were collected for bacteriological detection to ensure *P. multocida*-free status of the rabbits [12]. The sera of the rabbits were also tested for the presence of *P. multocida* IgG as described by Jaglic et al. [12].

Rabbits in groups of subcutaneous inoculation group and intranasal inoculation group were subcutaneously and intranasally inoculated with 6.0 × 10^4^ CFU of the isolate suspended in 100 μL of sterile normal saline, respectively. Rabbits in the negative control group were intranasally inoculated with 100 μL of sterile normal saline. The challenge does and routs were selected as described by Jaglic et al. [12, 23]. The clinical signs including cough, nasal discharge and dyspnea were monitored twice daily for 15 days for the all rabbits. At the end of the experiment, the gross lesions of the all rabbits were examined and tissue samples including tracheas, lungs, livers, hearts, spleens, kidneys and whole blood of all rabbits were collected for bacteriological examination, and the sera was used for serological examination as described by Jaglic et al. [12]. Lung samples from the rabbits of intranasal inoculation group were histologically examined using hematoxylin-eosin staining.

Prior to challenge, all the rabbits were anaesthetized with intravenous injection of ketamine (40 mg/kg). Rabbits that were caught up in the endpoint (dyspnoea, weight loss of 15% or inability to access feed or water), and rabbits that survived until the 15 days were also sacrificed under ketamine narcosis to minimize sufferings.

### Genome sequencing and comparative analysis

The whole genome of the isolate was sequenced using PacBio system (Pacific Biosciences, USA) at Shanghai Majorbio Bio-Pharm Technology Co., Ltd. (Shanghai, China). The PacBio system generated 244,686 total reads and 1,833,208,095 total bases, with the average reads length of 7492.08 bases and the average genome coverage of 891-fold. The raw reads were analyzed on the Majorbio Cloud Platform (www.majorbio.com), and then the complete genome of the isolate was de novo assembled. The complete genome of the isolate was annotated using the NCBI Prokaryotic Genome Annotation Pipeline. Genomic island and prophage were determined using IslandViewer 4 [32] and PHAST [18], respectively. Drug resistance genes were determined by comparing the sequence of the isolate against the comprehensive antibiotic resistance database (CARD) (https://card.mcmaster.ca/). The circular genome map of the isolate was plotted using DNAPlotter (version 17.0.1) [33]. The complete genomes of the *P. multocida* strains CQ2 (capsular serogroup A), HN06 (capsular serogroup D), Pm70 (capsular serogroup F), HN07 (capsular serogroup F), CIRMBP-0873 (capsular serogroup F) and CIRMBP-0884 (capsular serogroup F) were freely obtained from NCBI database under the accession numbers of CP033599, CP003313, AE004439, CP007040, CP020347 and CP020345, respectively. The whole genome comparison between the isolate and these strains was performed by using progressiveMauve alignment procedure on Mauve (version 2.4.0) [34], BLAST (version 2.4.0) [35], BRIG (version 0.95-dist) [36] and Easyfig (version 2.2.5) [37].

## Supplementary Information

**Additional file 1: Table 1.** The GIs in the genome of s4.

**Additional file 2: Table 2.** The prophages in the genome of s4.

**Additional file 3.** The sequence of the unique gene (LCY73_01710) identified in the s4 but not in the Pm70.

## Data Availability

The complete genome sequences of s4 were deposited in the NCBI GenBank (https://www.ncbi.nlm.nih.gov/genbank/) under the accession numbers of CP084165 (chromosome) and CP084164 (plasmid). The BioSample accession number is SAMN21591049, and BioProject accession number is PRJNA765870. Other raw datasets that support this study are available from the corresponding authors upon request, Wang J and Xie X.

## Abbreviations

*P. multocida*: *Pasteurella multocida*
RHDV: Rabbit hemorrhagic disease virus
*B. bronchiseptica*: *Bordetella bronchiseptica*
*S. aureus*: *Staphylococcus aureus*
PCR: Polymerase chain reaction
LPS: Lipopolysaccharide
BHI: Brain heart infusion
bp: base pair
CARD: Comprehensive Antibiotic Resistance Database
MLST: Multi-locus sequence typing
CFU: Colony forming units
GIs: Genomic islands
